# Silicon Nanosheets: An Emerging 2D Photonic Material with a Large Transient Nonlinear Optical Response beyond Graphene

**DOI:** 10.3390/nano12010090

**Published:** 2021-12-29

**Authors:** Michalis Stavrou, Aristeidis Stathis, Ioannis Papadakis, Alina Lyuleeva-Husemann, Emmanouel Koudoumas, Stelios Couris

**Affiliations:** 1 Department of Physics, University of Patras, 26504 Patras, Western Greece, Greece; m.stavrou@iceht.forth.gr (M.S.); a.stathis@iceht.forth.gr (A.S.); j.papadakis@iceht.forth.gr (I.P.); 2 Institute of Chemical Engineering Sciences (ICE-HT), Foundation for Research and Technology-Hellas (FORTH), 26504 Patras, Western Greece, Greece; 3 Institute for Nanoelectronics, Technical University of Munich, 80333 Munich, Germany; alina.lyuleeva@gmail.com; 4 Center of Materials Technology and Photonics, School of Engineering, Hellenic Mediterranean University, 71410 Heraklion, Crete, Greece; koudoumas@hmu.gr; 5 Department of Electrical and Computer Engineering, School of Engineering, Hellenic Mediterranean University, 71410 Heraklion, Crete, Greece

**Keywords:** silicon nanosheets, graphene nanosheets, transient nonlinear optical response, point defects, saturable absorption, Z-scan

## Abstract

The present work reports on the transient nonlinear optical (NLO) responses of two different types of 2D silicon nanosheets (SiNSs), namely hydride-terminated silicon nanosheets (SiNS–H) and 1-dodecene-functionalized silicon nanosheets (SiNS–dodecene). The main motivation of this study was to extend the knowledge regarding the NLO properties of these Si–based materials, for which very few published studies exist so far. For that purpose, the NLO responses of SiNS–H and SiNS–dodecene were investigated experimentally in the nanosecond regime at 532 and 1064 nm using the Z-scan technique, while the obtained results were compared to those of certain recently studied graphene nanosheets. SiNS–dodecene was found to exhibit the largest third-order susceptibility χ^(3)^ values at both excitation wavelengths, most probably ascribed to the presence of point defects, indicating the importance of chemical functionalization for the efficient enhancement and tailoring of the NLO properties of these emerging 2D Si-based materials. Most importantly, the results demonstrated that the present silicon nanosheets revealed comparable and even larger NLO responses than graphene nanosheets. Undoubtedly, SiNSs could be strong competitors of graphene for applications in 2D-material-based photonics and optoelectronics.

## 1. Introduction

Two-dimensional materials (2DMs) represent an emerging class of low-dimensional nanostructured systems, consisting of atomically thin sheets with strong covalent in-plane bonding and weak interlayer van der Waals bonding, both resulting in unique physicochemical properties [1,2]. Currently, 2DMs have stimulated considerable interest due to their potential applications in a plethora of scientific areas, including ultrafast lasing [3,4], optical limitation [5,6,7,8], catalysis [9], sensing [2,10], energy storage [11], and many more. The pioneer of 2DMs, graphene, has opened a new synthesis route for more 2D systems. Typical examples of 2DMs beyond graphene are xenes (e.g., silicene, phosphorene) [12,13], layered transition metal dichalcogenides (TDMTs, such as MoS_2_, MoSe_2_, WS_2_, WSe_2_) [14,15,16,17,18], metal carbides and nitrides (MXenes) [19], hexagonal boron nitride (h-BN) [20], and 2D hybrid perovskites [21,22].

In particular, the success of graphene has triggered many researchers to study other graphene-like 2DMs made up of other IV group elements (Si, Ge, Sn). Among them, the silicon-based counterpart of graphene, namely silicene, has gained great interest as it shares some of the outstanding properties envisaged for graphene, mainly due to the presence of Dirac fermions [23,24]. In addition, silicene has the extra advantage of being applicable in the silicon-based microelectronics industry [25], giving rise to great prospects for prominent electronic applications. In contrast to the perfectly planar geometry of graphene, silicene exhibits atomic-scale buckling indicative of the mixed sp^2^–sp^3^ nature of the hexagonal Si lattice [23,24]. It is expected that the distinct structure of silicene provides even better physical properties than those of graphene [26]. In addition, the mixed sp^2^–sp^3^ hybridized network of silicene gives an excellent reactive ability, facilitating its chemical functionalization. Therefore, the latter can be used as a powerful strategy to improve the poor stability and widen the zero-bandgap of silicene, factors that otherwise restrict its practical applications, especially in optoelectronics and photonics [27,28].

The main method for producing silicon nanosheets (SiNSs) involves epitaxial growth on various metallic substrates. To date, SiNSs have been successfully grown on Ag (1 1 1), Ir (1 1 1) and ZrB_2_ (1 1 1) substrates [29,30,31,32,33]. However, freestanding 2D silicene is inherently unstable and suffers from unwanted surface oxidation. On the other hand, the hydride-terminated SiNS (SiNS–H), the so-called silicane, is a more stable 2D SiNS if functionalized, for instance via a radical initiated hydrosilylation reaction. Specifically, hydrosilylation, which involves alkenes (e.g., 1-dodecene), is one of the most successful methods proposed to overcome the limitations of surface oxidation and the dispersibility of SiNSs [34], while it is also expected to allow the tailoring of the physicochemical properties of SiNSs. 

The strong optical nonlinearity and ultrafast responses are some of the key properties of layered materials, leading to their high potential in optoelectronics and photonics as optical limiters, saturable absorbers, optical modulators, and wavelength converters [35,36,37,38]. In this context, in 2009 it was found that graphene is an efficient atomic layer saturable absorber, which can be used in the field of ultrafast photonics to design pulse-shaping devices [39,40]. Regarding the practical demands, the newly synthesized 2DMs have always been a subject of interest, having been investigated by the nonlinear optics community. Among them, it is expected that graphene’s closely related material, silicene, can operate as successfully as graphene in several applications. However, there has not been much research work related to the NLO properties of silicene so far. In this context, the main scope of the current work is to shed light on the NLO properties of two different types of silicon nanosheets, namely hydride–terminated silicon nanosheets (SiNS–H) and 1-dodecene-functionalized silicon nanosheets (SiNS–dodecene) under 4 ns of visible (532 nm) and infrared (1064 nm) laser excitation using the Z-scan technique. The transient optical nonlinearities of the investigated SiNSs are determined and compared to those of graphene and other graphene-based nanosheets, all studied under similar excitation conditions.

## 2. Materials and Methods

### 2.1. Synthesis of SiNS–H and SiNS–Dodecene

**Figure 1:**
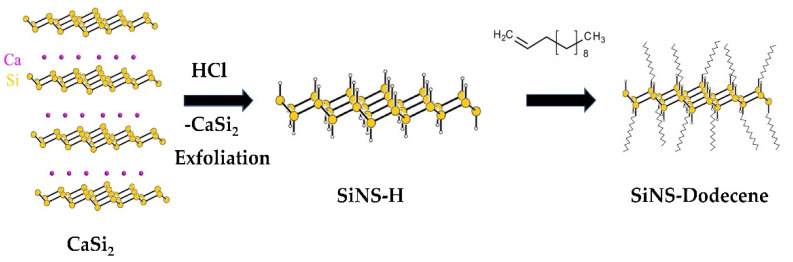
Schematic representation of SiNS–H synthesized by chemical exfoliation from CaSi_2_ and SiNS–dodecene obtained via hydrosilylation reaction with 1-dodecene.

The synthesis and surface modification of the silicon nanosheets were performed as previously described in detail elsewhere [41]. In brief, SiNS–H was directly synthesized through chemical exfoliation from the Zintl-phase calcium disilicide (CaSi_2_). After the deintercalation of calcium cations from the layered structure with aqueous HCl, the crystalline structure of CaSi_2_ collapsed, leading to the liberation of SiNS–H. The surface of the resulting hydride-terminating silicene was functionalized with 1-dodecene by utilizing hydrosilylation reactions to yield dodecyl–functionalized silicane (SiNS–dodecene). A schematic representation of the synthetic procedure used for SiNSs is presented in Figure 1.

### 2.2. Samples Preparation

SiNS–H and SiNS–dodecene were dispersed in different organic solvents; however, the toluene SiNSs dispersions showed greater uniformity. The prepared SiNSs toluene dispersions had a distinctive yellow color (turned to white or transparent upon oxidation) and were placed in 1-mm-thick quartz cells for the NLO measurements. Due to the strong oxophylicity of the Si atoms, the preparation of the SiNSs dispersions was performed inside a glove bag, while the cells containing the prepared samples were tightly sealed and were kept under argon atmosphere. Since the studied SiNSs precipitates relatively quickly in most of the common organic solvents, in turn forming agglomerates [42], the samples were left to rest for one day before the NLO measurements. Then, a part of the supernatant was transferred in the cells used for the NLO experiments. UV-Vis-NIR absorption spectra were taken systematically using a spectrophotometer to ensure the stability of the samples (concerning precipitation or oxidation). In all cases, the UV-Vis-NIR spectra did not exhibit any changes, suggesting that the dispersed SiNSs were not oxidized and remain stable during the experiments.

To safely determine the concentrations of the dispersions used for the Z-scan measurements, the dispersed mass of the sample was weighed for a given sample volume. In more detail, several dispersions were prepared in vials and then left to rest for 1 day. After this, some of the supernatant was collected and transferred into the cells. For lower concentrations, a measured volume of solvent was added into the 1 mm quartz cells. Then, the dispersions in the cells were monitored through UV-Vis-NIR absorption spectra to ensure that no precipitation occurred, i.e., the concentrations were stable. From these dispersions, a measured volume was collected and the concentration was determined via a TGA Q50 V6.7 Build 203 (Shimatzu, Kyoto, Japan). In more detail, the dispersion was heated in N_2_ atmosphere until the solvent was completely evaporated and the mass of the sample remained stable. This mass was then weighed using a high-precision scale and the concentration of the sample was determined.

### 2.3. Z-Scan Measurements

The determination of the third-order nonlinear optical (NLO) properties of SiNS–H and SiNS–dodecene was performed using the conventional single-beam Z-scan technique [43]. This technique was used as it has the great advantage of allowing the simultaneous evaluation of both the magnitude and sign of the nonlinear absorption and refraction of a sample from a single measurement. The aforementioned NLO quantities are expressed in terms of the nonlinear absorption coefficient *β* and nonlinear refractive index parameter *γ*′, respectively. The details of the Z-scan technique have been described in detail elsewhere [43,44], and only a brief description is presented here. According to this technique, the normalized transmittance of a sample mounted on a stepper motor and moving along the propagation z-direction of a focused laser beam is measured by means of two different experimental configurations, the so-called “open-aperture” (OA) and “closed-aperture” (CA) Z-scans. In the former transmission measurement, the transmitted sample laser light is totally collected by a large diameter lens and provides information about the nonlinear absorption. Simultaneously, in the latter measurement, i.e., the “closed-aperture” (CA) Z-scan measurement, the transmitted sample laser light is measured after having passed through a narrow aperture positioned in the far field, providing information on the sample’s nonlinear refraction. The OA Z-scan recording presents a transmission minimum or maximum, indicating reverse saturable absorption (RSA, corresponding to *β* > 0) or saturable absorption (SA, corresponding to *β* < 0), respectively. Correspondingly, the presence of a transmission valley-peak or peak-valley configuration in the CA Z-scan indicates self-focusing or self-defocusing behavior, respectively, corresponding to *γ*′ > 0 or *γ*′ < 0. If the nonlinear absorption is negligible, the nonlinear refractive index parameter *γ*′ can be directly deduced for the CA Z-scan, while in the presence of nonlinear absorption the nonlinear refractive index parameter *γ*′ is deduced from the so-called “divided” Z-scan obtained from the division of the CA Z-scan trace by the corresponding OA one.

The nonlinear absorption coefficient *β* is calculated by fitting the experimental OA Z-scan data points with Equation (1), while the nonlinear refractive index parameter *γ*′ can be determined by fitting the corresponding “divided” Z-scan with Equation (2).

(1) $$T\left(x\right)=\sum_{m=0}^{\infty }\frac{{\left(-\beta I_{0}L_{eff}\right)}^{m}}{{\left(1+x^{2}\right)}^{m}{\left(m+1\right)}^{3/2}}$$

(2) $$T\left(x\right)=1+\frac{4\gamma \prime kI_{0}L_{eff}x^{2}}{\left(1+x^{2}\right)\left(9+x^{2}\right)}$$

where *x* = *z*/*z*_0_, with z being the sample position and *z*_0_ the Rayleigh length; *I*_0_ is the on-axis peak irradiance; *L_eff_* is the sample’s effective length; *k* is the excitation wavenumber. 

The excitation laser sources employed for the Z-scan measurements were the fundamental and SHG outputs at 1064 nm and 532 nm, respectively, of a 4 ns Q-switched Nd–YAG laser system operating at a repetition rate between 1 and 10 Hz. However, during the experiments, the laser was operating at 1 Hz to prevent the manifestation of unwanted thermal or cumulative effects. For the experiments, the laser beam was focused into the sample by means of a 20 cm focal length quartz planoconvex lens. The beam spot radii at the focal plane were measured with a charge-coupled device (CCD) and was found to be 30 μm and 18 μm at 1064 nm and 532 nm, respectively. The laser beam energy was monitored by means of a calibrated joulemeter.

## 3. Results and Discussion

Both SiNS samples were characterized by atomic force microscopy (AFM), X-ray photoelectron spectroscopy (XPS), Fourier transform infrared (FTIR) spectroscopy, and thermogravimetry analysis (TGA). A detailed description of the characterization results has been given elsewhere [41]. Briefly, the AFM images showed that the thicknesses of SiNS–H and SiNS–dodecene were about 2.3 nm and 2.8 nm, respectively. According to theoretical calculations, the fully hydrogenated single-layered silicene (silicane) had a thickness of about 0.3–0.5 nm [24]. Therefore, it can be safely concluded that both samples exhibited multilayered structures. The FTIR spectra of SiNS–dodecene verified the successful functionalization of SiNS–H with 1-dodecene. The XPS data indicated that the dominant oxidation state of Si is 0. In addition, it has been shown that the functionalization with 1-dodecene yields ∼19% coverage of Si atoms with dodecene ligands. The latter is in tune with the estimation of the surface coverage obtained from the TGA analysis.

**Figure 2:**
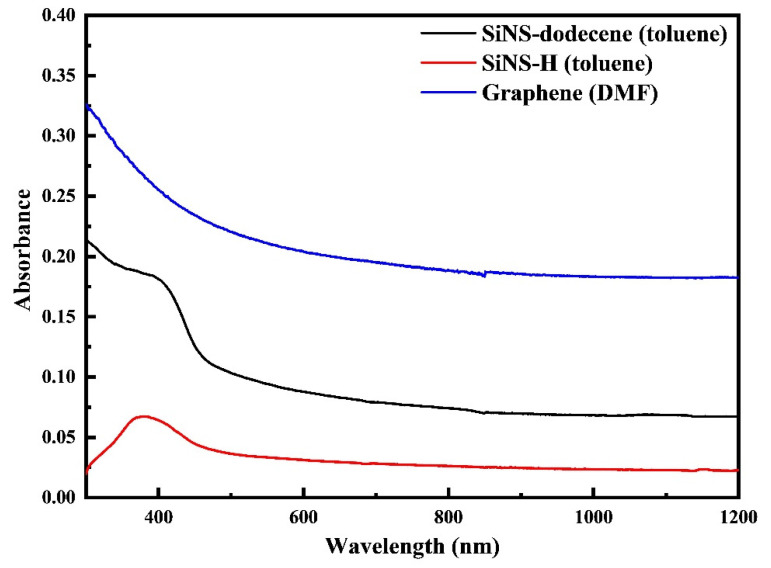
UV-Vis-NIR absorption spectra of SiNS–H and SiNS–dodecene toluene dispersions and a graphene dispersion in DMF. All spectra correspond to a concentration of 0.1 mg/mL.

In Figure 2, representative UV-Vis-NIR absorption spectra of the as prepared SiNS–H and SiNS–dodecene dispersions in toluene are presented, all referring to a concentration of 0.1 mg/mL. For comparison purposes, the UV-Vis-NIR absorption spectrum of a multilayered graphene (i.e., 2–5 layers) dispersed in dimethylformamide (DMF) with a concentration of 0.1 mg/mL is also included in Figure 2. As depicted, the absorption spectra of SiNSs appear smooth and featureless throughout the visible and near-infrared regions, exhibiting a characteristic absorption peak in the 350–400 nm spectral region, which is ascribed to transitions occurring from occupied σ to unoccupied σ* states [45]. An identical morphology can also be observed for the UV-Vis-NIR absorption spectrum of graphene (see e.g., Figure 2), where, in contrast to SiNSs, the 350 nm absorption is attributed to π–π* transitions of the aromatic C=C bonds [46].

As mentioned previously, silicene not only possesses a graphene-like structure but also exhibits most of the remarkable properties of graphene, providing an alternative 2D material. Therefore, it is reasonably expected that silicon nanosheets may exhibit comparable NLO properties to those of graphene. As a matter of fact, in a recent study by our group reporting on the ultrafast third-order nonlinearities of SiNS–H and SiNS–dodecene [41,47], it was revealed that SiNSs exhibited a comparable and even higher NLO response than that of graphene, emphasizing their efficiency for photonic and optoelectronic applications.

It is noteworthy at this point to mention that the physical processes and mechanisms underlying the NLO response of an atomic or molecular system are strongly dependent on the laser excitation or pulse duration that interacts with the medium [48,49]. For instance, under ultrafast laser excitation conditions (e.g., typically in the fs regime), the induced NLO response is exclusively attributed to nonlinearities arising from a purely electronic origin (instantaneous NLO response). On the other hand, when the excitation laser pulse duration is of several ns or longer, the NLO response is more transient than it is instantaneous. In this case, physical processes operating over a longer timescale occur, such as vibrational contributions, molecular orientation, free-carrier absorption, excited states populations, thermal lensing effects, or even a combination of the above, overwhelming the instantaneous electronic response. Then, because of the different mechanisms involved in the observed NLO response, the related NLO parameters are stated as effective quantities (e.g., effective third-order susceptibility χ^(3)^). To the best of our knowledge, the current work is the first experimental attempt to determine the effective NLO response of silicon nanosheets under 4 ns of visible (532 nm) and infrared (1064 nm) laser excitation and to compare the obtained findings with those of graphene nanosheets. For the accurate determination of the third-order susceptibility χ^(3)^ and the related NLO parameters (nonlinear absorption coefficient *β* and nonlinear refractive index parameter *γ*′) of both SiNS dispersions and to gain a deeper insight into the physical mechanisms underlying their transient NLO response, several Z-scans of different dispersions with concentrations ranging from 0.05 to 0.4 mg/mL and for a wide range of incident laser intensities (i.e., up to 72 and 110 MW/cm^2^ for visible and infrared laser excitation, respectively) were performed.

**Figure 3:**
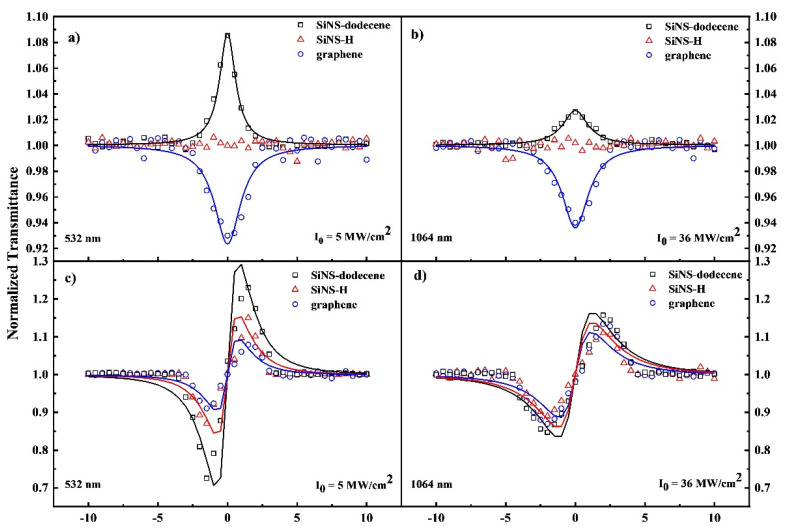
“Open-aperture” (**a**,**b**) and “divided” (**c**,**d**) Z-scans of SiNS–H, SiNS–dodecene, and graphene dispersions at 532 nm and 1064 nm for 4 ns. The concentration for all dispersions was 0.1 mg/mL.

In Figure 3, typical OA and “divided” Z-scans of SiNS–H and SiNS–dodecene dispersions at a concentration of 0.1 mg/mL are presented, obtained under 4 ns of 532 and 1064 nm laser excitation. For comparison purposes, the corresponding Z-scans of a 0.1 mg/mL graphene dispersion are also included in this figure. The solid lines represent the best fits of the experimental data (solid points) using Equations (1) and (2). Similar energy-dependent Z-scan recordings are depicted in Figure S1. To check for any significant contribution arising from the solvent, Z-scan measurements of the neat solvent were also performed under similar experimental conditions. However, within the range of incident laser intensities used, toluene did not reveal any measurable NLO response. As a result, the sign of the NLO absorption and refraction of SiNS–H and SiNS–dodecene can be straightforwardly evaluated from a simple inspection of the OA and divided Z–scan curves, as presented in Figure 3. As can be seen in Figure 3a,b, the OA Z-scans of SiNS–dodecene exhibited a transmission maximum in the focal plane (*z* = 0) under both excitation wavelengths, corresponding to a saturable absorption (SA, *β* < 0) behavior. The SA behavior of SiNS–dodecene is opposite to the reverse saturable absorption (RSA, *β* > 0) that can be observed for graphene under similar experimental conditions. This sign alternation of the nonlinear absorption coefficient *β* reflects the different operational physical processes underlying the NLO response of SiNSs and graphene under ns laser excitation. For example, the RSA behavior of graphene can be described in terms of two-photon absorption (TPA) or excited-state absorption (ESA) [49]. On the other hand, SiNS–dodecene displayed SA behavior for all dispersion concentrations and even for the highest laser intensities used (see Figure S1a,b) where the TPA is more likely to occur, as discussed elsewhere [50]. Interestingly, the corresponding OA Z-scans of SiNS–H were flat (see Figure 3a,b), suggesting negligible NLO absorption for the range of concentrations and laser intensities used.

**Figure 4:**
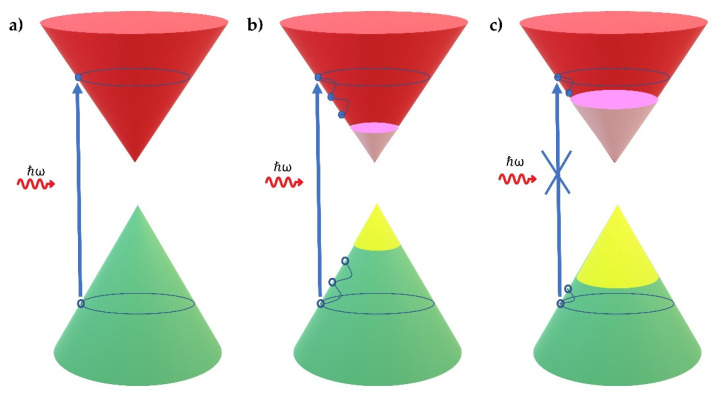
Absorption of photons in silicane: (**a**) schematic excitation process, where the arrow corresponds to an interband transition; (**b**) the photoexcited carriers thermalize and cool down to form a Fermi-Dirac distribution; (**c**) under high incident laser intensity, the photoexcited carriers occupy all the available states near the edge of the conduction and valence bands, thereby blocking any further absorption.

For SiNSs and graphene, electrons from the valence band can be promoted into the conduction band under sufficient laser intensity, while simultaneously empty states (i.e., holes) are generated in the valence band (Figure 4a). As for graphene, it is expected that the photoexcited electron–hole pairs of SiNSs will be converted to hot carriers, which shortly cool down and relax though carrier–carrier and electron–phonon scatterings forming a Fermi–Dirac distribution. In the following several ps, the hot carriers cool down rapidly through relaxation processes, including intraband phonon scattering and electron–hole recombination, thereby building an equilibrium electron and hole distribution (Figure 4b). For graphene, the former relaxation process has been reported to occur in the range of 70–150 fs after excitation, while the latter one occurs in the range of 0.5–2 ps [51,52]. Concerning the SiNS samples, in a recent study reporting on the ultrafast carrier dynamics of epitaxially grown silicene, it was verified that the previously mentioned relaxation processes take place in several tens to hundreds of fs and ps after photoexcitation, respectively [53]. The use of a sufficiently high incident laser intensity results in more efficient intraband transitions of charge carries. Therefore, all of the available states lying near the edges of the valence and conduction bands are occupied, blocking any further excitation of carriers due to the Pauli exclusion principle (Figure 4c). This situation implies the quenching of optical absorption being expressed as SA behavior and is known as Pauli Blocking [40]. This situation, i.e., the saturation of absorption, is a common feature in all graphene-like 2DMs, such as graphene and silicon nanosheets.

Typical characteristic “divided” Z-scans for SiNS–H and SiNS–dodecene under visible and infrared laser excitation are depicted in Figure 3c,d, all referring to the same concentration of 0.1 mg/mL. As shown, both SiNS samples displayed positive nonlinear refraction; that is, a valley-peak configuration, which indicates a self-focusing action (i.e., *γ*′ > 0), similar to the nonlinear refractive response observed for graphene under both excitation wavelengths (see Figure 3c,d). The energy-dependent “divided” Z-scans in Figure S1c–f show that the distance between the peak and the valley of normalized transmittance scales linearly with the incident laser intensity [43].

Then, following the standard procedures used for the analysis of the Z-scan measurements [43], the values of the NLO parameters were determined, i.e., the nonlinear absorption coefficient *β*, the nonlinear refractive index parameter *γ*′, and the effective third-order susceptibility χ^(3)^ of SiNS–H and SiNS–dodecene. The parameters summarized in Table 1, along with the corresponding values of graphene, were obtained under identical experimental conditions [54].

The obtained experimental findings unambiguously confirm that chemical functionalization via hydrosilylation reaction can be used to tune the NLO response of silicane. H-terminated silicene showed a greatly improved NLO response after appropriate chemical modification, as expressed by the enhanced third-order susceptibility *χ*^(3)^ of the 1-dodecene-modified SiNSs by factors of 2 and 1.5 for visible and infrared laser excitation, respectively. The possible mechanisms responsible for the enhancement of *χ*^(3)^ are described below.

Moreover, it is interesting to note the absence of any measurable NLO response for SiNS–H. However, the functionalization of silicane with 1-dodecene switched on a strong saturable absorption behavior compared to SiNS–H under both excitation wavelengths. This macroscopic on–off-like behavior can be ascribed to the existence of point defects introduced by the localized orbitals, containing an unpaired electron, the so-called “dangling bond” orbitals. More precisely, reports about some Si–H films and amorphous Si–H have shown that the point defects can create mid-gap states and local potential fluctuations that can lead to spatially localized energy states at the edges of valence and conduction bands, the so-called Urbach band tails [57,58,59]. Similarly, the chemical modification of SiNS–H may introduce these defect states because of the radical intermediates in the hydrosilylation mechanism [60]. These point defects could operate as trapping centers for the electrons of the conduction band. Therefore, the photo-excited electrons get trapped, resulting in prolonged relaxation time for these electrons from the conduction to valence band, meaning the incident photons cannot be absorbed due to the Pauli exclusion principle. Therefore, SiNS–dodecene has a higher probability of absorption bleaching, as expressed by its SA behavior, due to the presence of trapping centers.

Concerning the nonlinear refractive response of both SiNSs, in some other studies it has been reported that amorphous Si–H possesses a larger nonlinear refractive index parameter *γ*′ than crystalline Si [61,62]. The larger value of *γ*′ was ascribed to free carrier refraction, induced by two-step absorption due to the presence of defects states in amorphous Si–H [62]. In addition, it was shown that the nonlinear refractive parameter *γ*′ has the same sign as the Kerr effect, causing a refractive nonlinear response [62]. It is, therefore, reasonable to assume that the same nonlinear process could explain the enhanced refractive nonlinearities of SiNS–dodecene compared to those of SiNS–H under both excitation wavelengths. Therefore, the enhanced nonlinear refractive response observed in SiNS–dodecene may arise from a single photon resonance from mid-gap states, generated due to the existence of “dangling bond” orbitals, as mentioned previously. It should be added that both SiNS–H and SiNS–dodecene were found to exhibit much larger nonlinear refractions than that of graphene, especially under visible excitation. This finding is quite important, since it suggests that these silicon-based 2D materials, due to their high refractive nonlinearities (i.e., in the order of 10^−21^ m^2^/W), can be useful and promising for designing all-optical switching devices [63].

It is noteworthy that both silicon nanosheets exhibited ca. 1.5 (for SiNS–H) and 3 (for SiNS–dodecene) times larger NLO response under visible excitation than that of graphene. On the contrary, in the case of infrared excitation, graphene has been reported to exhibit a very similar NLO response to that of SiNS–dodecene and about double the NLO response of SiNS–H. Taking into account the negligible nonlinear absorptive response of SiNS–H, the larger third-order susceptibility of SiNS–H compared to graphene, especially under visible excitation, can be ascribed to the stronger Kerr effect. On the other hand, SiNS–dodecene exhibited an even stronger NLO response than graphene, most probably due to the presence of point defects. The present results indicate that both studied SiNSs can compete effectively with graphene, as they present comparable and even higher NLO responses than graphene, especially under visible excitation conditions. Concequently, SiNSs can eventually substitute graphene, being capable of serving better several photonic and optoelectronic applications.

Finally, the NLO properties of the present SiNSs will be compared to those of some graphene derivatives (as e.g., graphene oxide (GO), nitrogen-doped GO (N-GO), boron–doped GO (B-GO) and fluorographene (CF)), which have been recently investigated under similar excitation conditions [5,55,56]. For comparison purposes, all the obtained results are summarized in Table 1. As can be seen from this table, the presently studied SiNSs were found to exhibit the largest third-order susceptibility *χ*^(3)^ among these 2D systems. These findings confirm that a new era of 2D materials such as graphene has already started in terms of advanced nanotechnology devices, since 2D silicon-based materials could eventually replace graphene nanosheets in several optoelectronic and photonic applications.

## 4. Conclusions

Summarizing, the nonlinear absorption and refraction of two different silicon nanosheets, namely SiNS–H and SiNS–dodecene, were studied employing 532 and 1064 nm laser pulses for 4 ns. The present results revealed that the magnitude of the NLO response of silicon nanosheets is comparable and even higher than that of graphene nanosheets, especially under visible excitation. In addition, the functionalization of the hydride-terminated silicon nanosheets with 1-dodecene resulted in an important enhancement of its nonlinear refractive response and a noticeable “switch on” effect of the NLO absorptive response, most probably due to the presence of point defects. The present findings demonstrate the great potential of silicon nanosheets and that they can efficiently compete with graphene being very promising candidates for next-generation photonic and optoelectronic applications. 

## Figures and Tables

**Table 1:** NLO parameters of SiNS–H, SiNS–dodecene, and graphene (G) determined under 532 nm and 1064 nm laser excitation for 4 ns.

λ (nm)	Sample	*β* (×10^−11^ m/W)	*γ*’ (×10^−1^^8^ m^2^/W)	|*χ*|^(3)^ (×10^−13^ esu)
532	SiNS–H in toluene	-	1990 ± 360	2820 ± 520
	SiNS–dodecene in toluene	−1243 ± 140	4014 ± 540	5724 ± 764
	G in DMF ^a^	1240 ± 149	1440 ± 173	2000 ± 313
	GO in H_2_O ^b^	43.5 ± 4.0	−81 ± 8	93 ± 9
	N–GO in DMF ^c^	453 ± 70	235 ± 32	388 ± 52
	B–GO in DMF ^c^	524 ± 52	304 ± 32	481 ± 48
	CF in DMF ^d^	601 ± 72	−1265 ± 177	1769 ± 230
1064	SiNS–H in toluene	-	297 ± 40	423 ± 56
	SiNS–dodecene in toluene	−110 ± 21	425 ± 80	621 ± 127
	G in DMF ^a^	526 ± 65	401 ± 54	778 ± 96
	GO in H_2_O ^b^	-	-	-
	N-GO in DMF ^c^	331 ± 40	229 ± 38	344 ± 54
	B-GO in DMF ^c^	429 ± 35	254 ± 38	398 ± 51
	CF in DMF ^d^	100 ± 17	−193 ± 28	270 ± 39

^a^ Values taken from ref. [54]. ^b^ Values taken from ref. [55]. ^c^ Values taken from ref. [5]. ^d^ Values taken from ref. [56].

## Data Availability

Not applicable.

## Supplementary Materials

The following supporting information can be downloaded at: https://www.mdpi.com/article/10.3390/nano12010090/s1: Figure S1: Energy-dependent (a,b) OA and (c,d) “divided” Z-scans of SiNS–dodecene and similar (e,f) “divided” Z-scans of SiNS–H under 532 nm laser excitation for 4 ns. The concentration for all dispersions was 0.1 mg/mL.
