# Supplementary material for: Silicon Nanosheets: An Emerging 2D Photonic Material with a Large Transient Nonlinear Optical Response beyond Graphene
Source: Nanomaterials (Basel). 2021 Dec 29;12(1):90. doi: 10.3390/nano12010090 (PMC8746558; doi:10.3390/nano12010090)
Supplement: Supplementary file 1 [file nanomaterials-12-00090-s001.zip › nanomaterials-1499434-supplementary.pdf]

Supporting Information

Silicon Nanosheets: An Emerging 2D Photonic Material with a Large Transient Nonlinear Optical Response beyond Graphene

Michalis Stavrou [1,2], Aristeidis Stathis [1,2], Ioannis Papadakis [1,2], Alina Lyuleeva-Husemann [3], Emmanouel Koudoumas [4,5] and Stelios Couris [1,2,*]

[1] Department of Physics, University of Patras, 26504 Patras, Greece; m.stavrou@iceht.forth.gr (M.S.); a.stathis@iceht.forth.gr (A.S.); j.papadakis@iceht.forth.gr (I.P.)
[2] Institute of Chemical Engineering Sciences (ICE-HT), Foundation for Research and Technology-Hellas (FORTH), 26504 Patras, Greece
[3] Institute for Nanoelectronics, Technical University of Munich, 80333 Munich, Germany; alina.lyuleeva@gmail.com
[4] Center of Materials Technology and Photonics, School of Engineering, Hellenic Mediterranean University, 71410 Heraklion, Crete, Greece; koudoumas@hmu.gr
[5] Department of Electrical and Computer Engineering, School of Engineering, Hellenic Mediterranean University, 71410 Heraklion, Crete, Greece
* Correspondence: couris@iceht.forth.gr or couris@upatras.gr; Tel.: +30-2610996086

Energy-depedent Z-scans of SiNS–H and SiNS–dodecene toluene dispersions

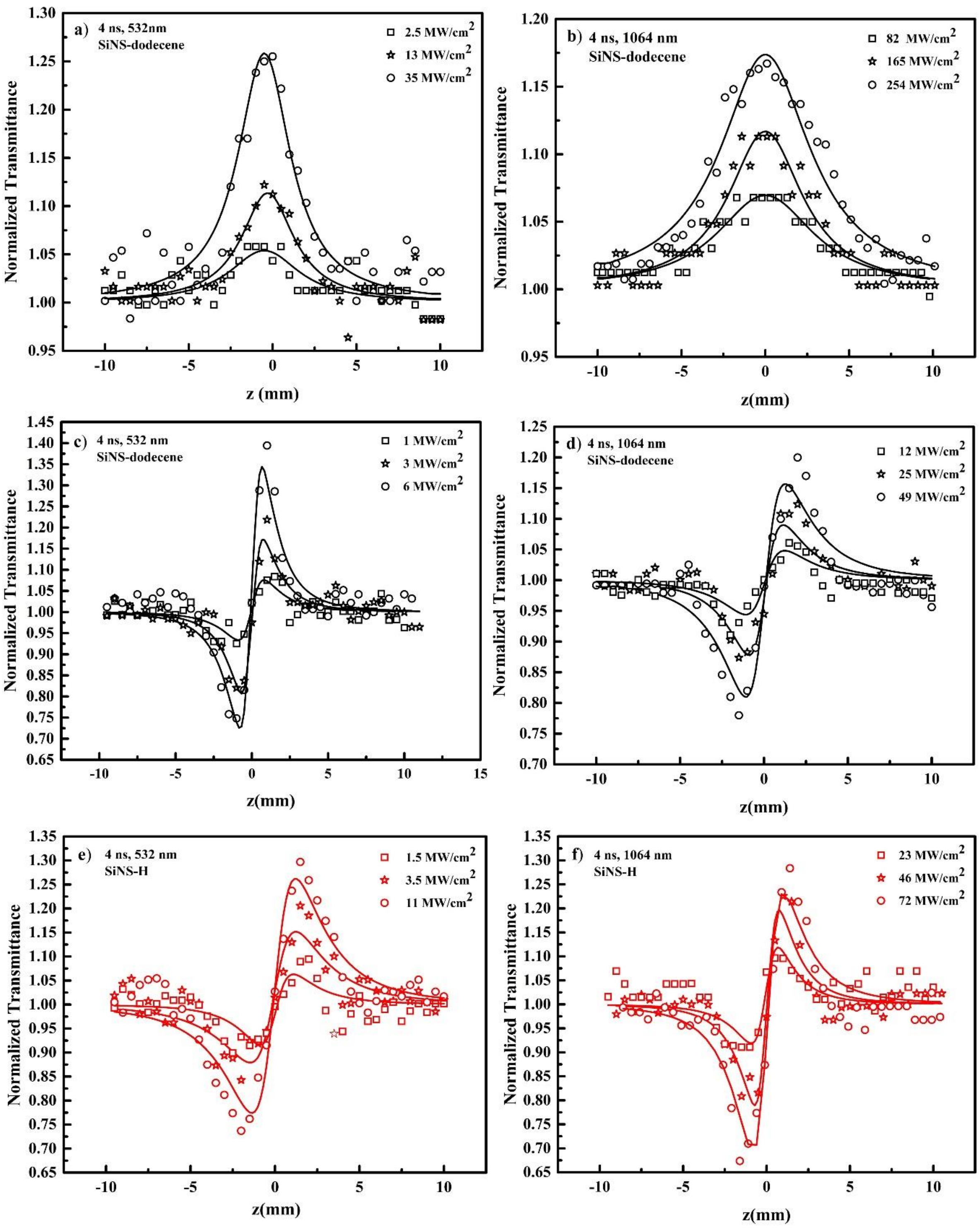

Figure S1. Energy-dependent (**a**,**b**) OA and (**c**,**d**) "divided" Z-scans of SiNS-dodecene and similar (**e**,**f**) "divided" Z-scans of SiNS–H under 4 ns, 532 nm laser excitation. The concentration of all dispersions was 0.1 mg/mL.
